# A Notice of the Late Frederick Turnpenny, M. D.

**Published:** 1840-06-06

**Authors:** 


### A NOTICE OF THE LATE FREDERICK TURNPENNY, M. D.

[Communicated for the Medical Examiner.]

Died, on the evening of the 2d inst, in the 31st year of his age, Frederick Turnpenny, M. D.

The brief career of this estimable physician was distinguished by a force of intellect, and an enthusiastic devotion to professional pursuits, which gave promise of future eminence and usefulness.

Being destined by his friends for a pharmaceutist, Dr. Turnpenny was apprenticed at an early age to a respectable apothecary, and acquired with extraordinary facility and rapidity, not only a practical acquaintance with the art, but a thorough scientific knowledge of materia medica and pharmacy. At the age of 20 he was suffered to abandon his pursuit, and to devote himself to the study of medicine: he accordingly entered as a pupil with the late lamented Dr. Parrish, for whom he entertained the warmest esteem and affection. He had long cherished a desire to pursue medical studies, and entered upon the task with a degree of energy and enthusiasm, which did not forsake him during the whole course of his pupilage.

He graduated at the University of Pennsylvania in the year 1832, being conspicuous amongst his fellows for rare medical attainments, and superior talents. He commenced practice in Philadelphia this year, and soon found ample opportunity for the exercise of his skill, by the appearance of the epidemic cholera. He was chosen by Dr. Jackson as one of his assistants in the cholera hospital under his direction, and signalized himself by his skilful and unremitting attentions to the duties of this station.

His success as a practitioner was equal to his most sanguine expectations. Few men have risen more rapidly in public confidence and esteem, or have been better able to sustain the rank which may have been assigned them by partial friends. Dr. Turnpenny possessed a native aptitude for the profession, which, combined with a highly pleasing address, and with the most persevering and assiduous attention to every professional duty, constituted the basis of a reputation, which, had his life been prolonged, would doubtless have placed him in the front rank of his profession.

Unlike many physicians who become absorbed in practical duties, Dr. Turnpenny devoted much of his leisure time to study, keeping himself informed of every new discovery and improvement which the indefatigable researches of medical inquirers are constantly unfolding. He devoted himself with peculiar assiduity to the study of the institutes of medicine, and was a warm admirer of the writings of many of the French authors, who, while they have developed much that is erroneous and speculative, have still shed new light over many obscure and heretofore unexplored regions of our science. He was a zealous advocate of the doctrines of the phrenological school, and looked to the science of phrenology as a means whereby many of the phenomena of the diseases of the brain, which are now exceedingly obscure, or altogether inexplicable, would at some future period be satisfactorily explained. So well informed was Dr. Turnpenny in the details of this science, that he was selected by Dr. Morton to furnish an article on the phrenological developments of the different races of men, to be incorporated in his splendid work, Crania Americana: he was prevented, however, from executing his task by the invasion of disease.

In connexion with his researches on diseases of the brain, he published, several years since, a highly ingenious paper on the pathology of epilepsy, in the treatment of which intractable malady he had met with unusual success.

During the summer of 1838 Dr. Turnpenny was seized with an affection of the bowels, attended with acute pain and frequent discharges; the remedies addressed to the disease failed in producing permanent relief, and the energies of his system became greatly impaired.

After suffering for a year with the disease, he was forced reluctantly to abandon his profession, and to seek relief by a voyage to England. This expedient, from which much benefit was expected by his friends, seemed only to render his case more alarming. Soon after getting to sea, the bowel affection subsided, and was followed by symptoms indicating the development of meningitis.

He became the subject of treatment for this affection on his arrival in England; and when he had so recovered as to render exertion admissible, he was diligently employed in seizing every opportunity whereby he could add to his stock of medical knowledge. At the time of his embarkation for this country, Dr. Turnpenny had so far regained his health as to induce the hope of his entire restoration. On the return voyage, however, his former symptoms were renewed; and very soon after his arrival he was seized with an attack of acute meningitis, which terminated his existence after an illness of sixteen days.

Agreeably to his oft-repeated request, a post mortem examination was instituted in the presence of several of his medical friends. The appearances, on dissection, indicated chronic meningitis, with decided opacity and thickening of the membranes, principally over the superior portion of the middle and anterior lobes of the cerebrum, and at the base of the cerebellum; slight lymphatic deposits were observed between the membranes at several points, with considerable serous effusion beneath the membranes and in the cavities of the ventricles. The substance of the brain presented a perfectly healthy appearance, the convolutions being unusually deep.

The mucous membrane of the colon, throughout the greater portion of its surface, was thickened, and its follicles enlarged, exhibiting evidences of chronic inflammation without ulceration, which appeared to those present to have been in the progress of recovery at the time of the patient's death. Several cicatrices, filled with calcareous deposit, were found in the upper lobe of the right lung, and one in the left,—an appearance which was the more remarkable, as no suspicion had existed of the patient having at any period been the subject of phthisis pulmonalis. The other portions of the lungs were perfectly healthy. The mesenteric glands were considerably enlarged; other viscera sound as far as examined.

In the early demise of Dr. Turnpenny, his medical brethren have lost the services of a member of their body who had devoted his life to the advancement of science, and to the support of the dignity and honour of his profession. No sordid or selfish views ever permitted him to swerve from the path of integrity, or to compromise those high principles of medical ethics which bind the medical fraternity into one harmonious body.

That one so fitted by nature and education to advance the interests of our science, should be cut off, in the very morning of existence, when his labours were just beginning to be valuable, is truly a subject of general regret. While amidst the circle of his immediate associates, regret deepens into sorrow for the loss of a friend, for whose amiable deportment, and manly virtues, they will long cherish the most lively recollection.

